# Deep Eutectic Solvents as Active Pharmaceutical Ingredient Delivery Systems in the Treatment of Metabolic Related Diseases

**DOI:** 10.3389/fphar.2021.794939

**Published:** 2021-12-24

**Authors:** Cixin Huang, Xiunian Chen, Chao Wei, Hongwei Wang, Hua Gao

**Affiliations:** ^1^ State Key Laboratory Cultivation Base, Shandong Provincial Key Laboratory of Ophthalmology, Eye Institute of Shandong First Medical University, Qingdao, China; ^2^ Eye Hospital of Shandong First Medical University (Shandong Eye Hospital), Jinan, China; ^3^ Medical College, Qingdao University, Qingdao, China; ^4^ School of Ophthalmology, Shandong First Medical University, Jinan, China

**Keywords:** deep eutectic solvents, drug delivery system, metabolic diseases, cancer, diabetes mellitus, atherosclerosis

## Abstract

Metabolic related diseases such as cancer, diabetes mellitus and atherosclerosis are major challenges for human health and safety worldwide due to their associations with high morbidity and mortality. It is of great significance to develop the effective active pharmaceutical ingredient (API) delivery systems for treatment of metabolic diseases. With their unique merits like easy preparation, high adjustability, low toxicity, low cost, satisfactory stability and biodegradation, deep eutectic solvents (DESs) are unarguably green and sustainable API delivery systems that have been developed to improve drug solubility and treat metabolic related diseases including cancer, diabetes mellitus and atherosclerosis. Many reports about DESs as API delivery systems in the therapy of cancer, diabetes mellitus and atherosclerosis exist but no systematic overview of these results is available, which motivated the current work.

## Introduction

Metabolic related diseases are diseases caused by obstacles in the process of anabolism and catabolism in human body (Hoffman et al., 2021). Cancer, diabetes mellitus and atherosclerosis are the common types of metabolic diseases (Boroughs & DeBerardinis, 2015; Taylor et al., 2018; Poznyak et al., 2020), which impair metabolic homeostasis and are accompanied by a cluster of metabolic syndromes (Taylor et al., 2018). However, the traditional chemotherapy approaches were bounded in the treatment of metabolic diseases owing to the disadvantages of non-selectivity, low bioavailability and drug resistance (Xin et al., 2017; Pearson, 2019; Shi et al., 2019). Thus, it is becoming a new therapeutic strategy by combined use of traditional drugs with vigorous drug delivery systems for therapy of metabolic related diseases.

**GRAPHICAL ABSTRACT:**
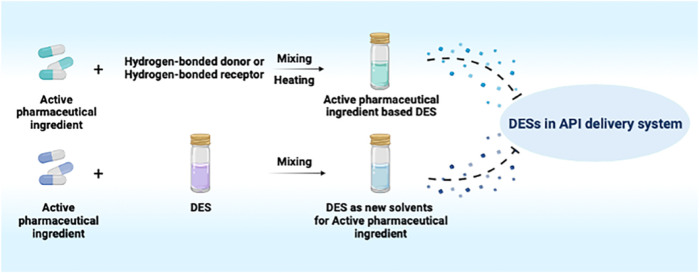


Drug delivery systems, such as liposomes, hydrogels, nanomaterials and ionic liquids, are significant alternatives to improve the solubility of poorly water-soluble active pharmaceutical ingredients (APIs) (De Jong & Borm, 2008; Edupuganti et al., 2012; Tian et al., 2020). Notably, ionic liquids (ILs) are widely used as green solvents in the pharmaceutical field because of their high polarity, non-reactivity to water, low vapor pressure, chemical and thermal stabilities (Sangtarashani et al., 2020). As a special kind of ILs, deep eutectic solvent (DES) is a mixture constituted with a certain proportion of hydrogen-bonded receptors (quaternary ammonium salt) and hydrogen-bonded donors (amides, amino acids, carboxylic acids and polyols) which has drawn increasing attention from researchers (Pena-Pereira & Namieśnik, 2014). Owing to their unique advantages, such as easy preparation, high adjustability, low toxicity, low cost, satisfactory stability and biodegradation, DESs have gradually become indispensable participants in the field of biomedicines (Gurkan et al., 2019; Liu et al., 2019).

**FIGURE 1:**
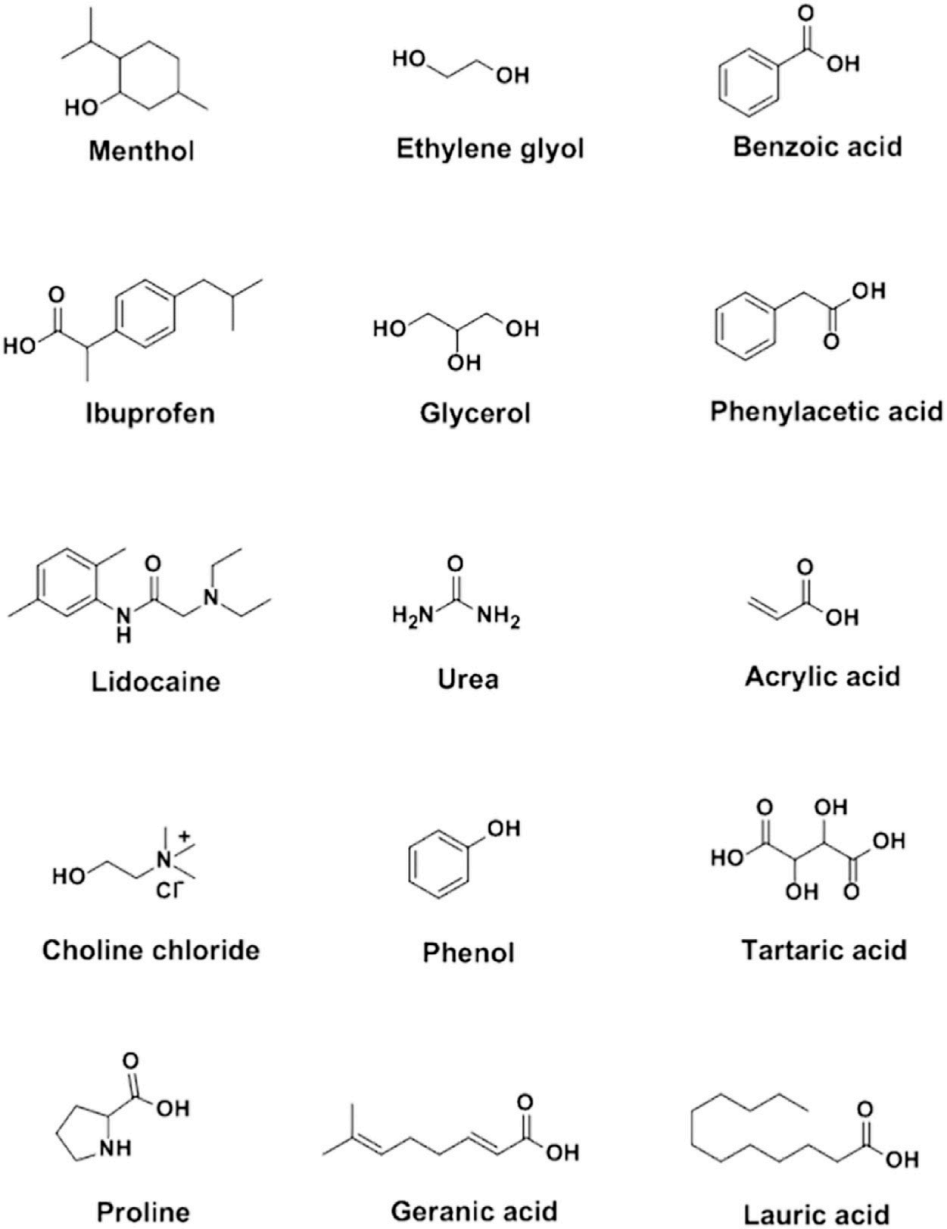
Chemical structures of the general components in synthesis of DESs.

Sekiguchi and Obi introduced the earliest eutectic mixture (sulfathiazole:urea based DES) into biomedical field in 1964 (Sekiguchi et al., 1964). The DES distinctly improved the solubility and oral absorption rate of sulfathiazole. Following this, lidocaine:procaine, ibuprofen:terpene and some other DESs have been developed as therapeutic DESs in the treatment of diseases (Stott et al., 1998; Wojnarowska et al., 2018). Besides, choline chloride:malic acid and choline bicarbonate:vanillic acid, were also synthesized and applied as excellent carriers to increase the solubility of unstable drugs (Zakrewsky et al., 2016; Shekaari et al., 2017), which is beneficial to drug storage and delivery. Carboxylic compounds, including benzoic acid, phenylacetic acid, lauric acid, myristic acid, acetylsalicylic acid, stearic acid, mandelic acid and maleic acid were commonly selected as DES components due to their low toxicity and stable hydrogen bonding capacity (Makoś et al., 2018; Pereira et al., 2019; Saha et al., 2020) (Figure 1).

Previously, DESs have been successfully used in the therapy of metabolic related diseases. However, there was no systematic review on these studies. Herein, we summarized the applications of DESs as API delivery systems in the treatment of metabolic diseases including cancer, diabetes mellitus and atherosclerosis.

## Classification of DESS in API Delivery Systems

### API-based DESs

APIs, such as menthol, ibuprofen, lidocaine, itraconazole, cannabidiol and testosterone, were involved in the formation of API-based DESs, which maintained in the therapeutic effect of the APIs (Aroso et al., 2015; Aroso et al., 2016; Cordeiro et al., 2017; Duarte et al., 2017; Gala et al., 2015; Kaplun-Frischoff & Touitou, 1997; Lodzki et al., 2003; Morimoto et al., 2000; Park et al., 2012; Silva et al., 2019; Stott et al., 1998; Wang et al., 2017; Woolfson et al., 2000) (Figure 1, Table 1). Silva et al. constructed hydrophobic DESs based on menthol and saturated fatty acids with different chain lengths, including stearic acid, myristic acid and lauric acid, and evaluated the toxicity and antibacterial activity of these DESs to human immortal keratinocyte line (HaCaT) cells (Silva et al., 2019). The results revealed that the synergistic effect of menthol and stearic acid on enhancing antibacterial activity and promoting wound healing, whereas the whole mixture exhibited no cytotoxicity, demonstrating the promising application of menthol:stearic acid based DESs in wound healing. Three DESs, prepared by complexation of APIs (i.e., ibuprofen, benzoic acid and phenylacetic acid) with menthol, possessed the characteristics of high solubility and high permeability, improving the bioavailability of the APIs (Duarte et al., 2017). In another study, seven terpenoids, including L-menthol, LD-menthol, d-limonene, l-menthone, 1,8-cineole, thymol and cymene were selected to be mixed with ibuprofen to prepare the DES system (Stott et al., 1998). Among them, the combination of ibuprofen and thymol maximizes permeability and produces a synergistic effect in penetration experiment.

**TABLE 1:** The composition, molar ratio and application of API-based DESs.

DESs composition	Molar ratio	API	Application	References
Menthol: Stearic acid	8:1	Menthol	Anti-microbial	Silva et al. (2019)
			Wound healing	
Menthol: Myristic acid	8:1	Menthol	Anti-microbial	Silva et al. (2019)
			Wound healing	
Menthol: Lauric acid	4:1	Menthol	Anti-microbial	Silva et al. (2019)
			Wound healing	
Menthol: Acetylsalicylic acid	1:1	Menthol	Anti-microbial	Aroso et al. (2016)
			Dissolution enhancer	
Menthol: Benzoic acid	3:1	Menthol	Anti-microbial	Duarte et al. (2017)
			Dissolution enhancer	
Menthol: Phenylacetic acid	2:1; 3:1	Menthol	Anti-microbial	Duarte et al. (2017)
			Dissolution enhancer	
Ibuprofen: L-Menthol	1:1	Ibuprofen	Anti-inflammatory; Penetration enhancer	Stott et al. (1998)
Ibuprofen: LD-Menthol	1:1; 7:13; 1:4	Ibuprofen	Anti-inflammatory; Penetration enhancer	Stott et al. (1998)
Ibuprofen: Thymol	2:3	Ibuprofen	Anti-inflammatory; Penetration enhancer	Stott et al. (1998)
Ibuprofen: 1,8-Cineole	2:3	Ibuprofen	Anti-inflammatory; Penetration enhancer	Stott et al. (1998)
lidocaine: Camphor	1:1	Lidocaine	Topical anesthesia	Gala et al. (2015)
			Penetration enhancer	
lidocaine: Tetracaine	1:1	Lidocaine	Topical anesthesia	Gala et al. (2015)
			Penetration enhancer	
Itraconazole: Phenol	1:1	Itraconazole	Anti-microbial	Park et al. (2012)
			Penetration enhancer	
Cannabidiol: Phosphatidylcholine	--	Cannabidiol	Anti-inflammation; Penetration enhancer	Lodzki et al. (2003)
Ibuprofen: Methyl nicotinate	1:1	Ibuprofen; Methyl nicotinate	Anti-inflammatory; Penetration enhancer	Woolfson et al. (2000)
Ibuprofen: Menthol	1:3	Ibuprofen; Menthol	Anti-inflammatory; Anti-microbial	Aroso et al. (2015)
Ibuprofen: Limonene	1:4	Ibuprofen; Limonene	Anti-inflammatory; Anti-cancer	Pereira et al. (2019)
Paeonol: Menthol	5:5	Paeonol; Menthol	Anti-microbial; Penetration enhancer	Wang et al. (2017)
Testosterone: Menthol	1:4	Testosterone; Menthol	Anti-microbial; Penetration enhancer	Kaplun et al. (1997)

Saturated fatty acid, menthol or ibuprofen was gently mixed with limonene to prepare dual-drug participating DESs by Pereira and his colleagues (Pereira et al., 2019). After a series of characterizations, it was found that the liquid DES could be formed using the ibuprofen:limonene mixture (molar ratio, 1:4) at room temperature or close to physiological temperature. Moreover, DESs can reduce the cytotoxicity of limonene, increase the solubility of ibuprofen, and exhibit anticancer and anti-inflammatory activities. Flurbiprofen:menthol based and ibuprofen:menthol based DESs could be included in mesoporous silica matrixes and 3D porous biopolymers, respectively, to obtain amorphous composites as API delivery systems with enhanced solubility of menthol (Morimoto et al., 2000; Cordeiro et al., 2017).

### DESs as New Solvents for APIs

#### Choline Chloride Based DESs

Choline chloride was widely used as a component in the synthesis of DESs (Alizadeh et al., 2020; Jeliński et al., 2019; Li & Lee, 2016; Mano et al., 2017; Morrison et al., 2009; Saha et al., 2020; Silva et al., 2019; Wikene et al., 2015; Zainal-Abidin et al., 2020) (Figure 1, Table 2). Choline chloride:glycolic acid, choline chloride:glycolic acid:oxalic acid, choline chloride:urea and choline chloride:malonic acid based DESs have been synthesized to increase solubility of low-soluble compounds including piroxicam, lidocaine, posaconazole, benzoic acid, danazol, griseofulvin, AMG517 and itraconazole (Morrison et al., 2009; Li & Lee, 2016). In 2016, Aroso and his colleagues synthesized DESs using choline chloride coupled with acetylsalicylate, benzoic acid and phenylacetic acid (Aroso et al., 2016). This indicates that organic acids can participate in the synthesis of DESs with choline chloride.

**TABLE 2:** The composition, molar ratio and API solubility of DESs as new solvents for APIs.

DES composition		Molar ratio	API	Solubility (mg/ml)	References
**Choline chloride**	Glycolic acid	1:2	Itraconazole	6.70	Li et al. (2016)
			Piroxicam	9.90	
			lidocaine	100.60	
			Posaconazole	76.80	
	Glycolic acid: Oxalic acid	1:1.7:0.3	Itraconazole	46.40	Li et al. (2016)
			Piroxicam	3.10	
			lidocaine	295.40	
			Posaconazole	88.40	
			Benzoic acid	23.00	
			Danazol	0.016	
	Urea	1:3	Griseofulvin	0.0061	Morrison et al. (2009)
			AMG517	0.00022	
			Itraconazole	<0.001	
			Benzoic acid	18.0	
	Malonic acid	1:3	Danazol	0.1007	Morrison et al. (2009)
			Griseofulvin	0.0044	
			AMG517	0.014	
			Itraconazole	6.60	
	Glycerol	1:1	Curcumin	7.25	Jeliński et al. (2019)
	Maleic acid	3:1	Curcumin	0.0667	Wikene et al. (2015)
	1,2-Propanediol	1:2	Aspirin	202.00	Lu et al. (2016)
			acetaminophen	324.00	
			Naproxen	45.26	
	Levulinic acid	1:2	Ketoprofen	329.10	Lu et al. (2016)
**Proline**	Urea	2:1	Berberibe	12.3	Sut et al. (2017)
	Malic acid: Lactic acid: Water	1:0.2:0.3:0.5	Berberibe	25.0	Sut et al. (2017)
	Glutamic acid	2:1	Rutin	2.9384	Faggian et al. (2016)
	Choline chloride	1:3	Rutin	2.7992	Chen et al. (2015)
**Others**	Borneol:Menthol	1:3	Daidzein	0.00031	Shen et al. (2011)
	Glucose:Sucrose	1:1	Curcumin	0.05211	Wikene et al. (2015)
	Choline bicarbonate: Geranic acid	1:1	Insulin	--	Banerjee et al. (2017)
	Choline bicarbonate: Geranic acid	1:1	BSA	--	Banerjee et al. (2017)
	Camphor:Menthol	1:1	Ibuprofen	282.11	Myung et al. (2012)

In a study conducted by Saha et al., the atomic-level interaction of choline chloride and acetylsalicylic acid in DESs was investigated by combining molecular dynamics, density functional theory, Raman spectroscopy and IR spectroscopy (Saha et al., 2020). Their results show that the electrostatic attraction of cations-anions was destroyed, while highly interconnected hydrogen bonds were formed between choline chloride and acetylsalicylic acid in DESs, which is similar to the characteristics of choline chloride:urea, choline chloride:ethylene glycol and choline chloride:glycerol (Figure 1). The non-bonding interaction between choline chloride and acetylsalicylic acid plays an important role in the formation of DES at room temperature. In addition, choline chloride:glycerol exhibit 12,000-fold higher solubility toward curcumin than aqueous solution at room temperature (Jeliński et al., 2019), demonstrating the potential of the choline chloride:glycerol-based DES as an effective delivery system for the dissolution of curcumin.

In another study, a fast-dissolving delivery system was designed by encapsulation of the gelatin fiber with choline chloride:mandelic acid based DES. Maintaining the antibacterial activity of mandelic acid, the system has no cytotoxicity effect in mouse lung fibroblasts cell line L929 and exhibits a strong resistance in Gram-positive bacteria and Gram-negative bacteria (Mano et al., 2017). Furthermore, a kind of molecularly imprinted polymer using choline chloride:caffeic acid:ethylene glycol (molar ratio, 1:0.4:1) was prepared and used for the molecular recognition of polyphenols (Silva et al., 2019), indicating the potential of ternary DESs based molecularly imprinted polymers in the separation, purification and delivery of the drug (Figure 1). Furthermore, ternary DESs, choline chloride:sucrose:water (molar ratio, 4:1:4) and choline chloride:glycerol:water (molar ratio, 1:2:1), can be used as functionalized agents for modification of graphene (Zainal-Abidin et al., 2020). DES-functionalized graphene possesses distinct electrical, mechanical and optical properties from the pristine graphene. Afterwards, adriamycin was loaded onto DES functionalized graphene, suggesting that choline chloride based DESs could act as promising green functionalized agents for the modification of nano-drug carriers.

#### Amino Acid Based DESs

The amino acid based DESs have good solubility to some specific compounds (Chen et al., 2015; Faggian et al., 2016; Liu et al., 2018; Sut et al., 2017) (Figure 1, Table 2). As non-toxic green natural DESs, amino acid based DESs are mainly food grade, and the solubility of the compounds in the DESs can be changed by adjusting the water content (Liu et al., 2018). The *in vivo* administration of berberibe was investigated using amino acid based DESs, including proline:malic acid (molar ratio, 2:1), proline:urea (molar ratio, 2:1) and proline:malic acid:lactic acid:water (molar ratio, 1:0.2:0.3:0.5) (Sut et al., 2017). The results indicated that proline:malic acid and lactic:proline:malic acid:water possessed better solubility to berberibe than ethanol and water. Meanwhile, *in vivo* experiments have demonstrated that proline based DESs can significantly increase the bioavailability of berberibe. In addition, proline:glutamic acid (molar ratio, 2:1) was used to explore the potential of DES as a solubilizing agent for polyphenol administration (Chen et al., 2015; Faggian et al., 2016). Take rutin, one of the most famous flavonoids, for example, the solubility of rutin in amino acid based DESs is 20-fold higher than that in water, which greatly expands its pharmacological applications. Furthermore, citric acid:l-arginine:water (molar ratio, 1:1:7) can be used to increase the solubility of ethambutol.

#### Others

It has been reported that a borneol:menthol (molar ratio, 1:3) based DES delivery system can promote the intestinal absorption of daidzein (Shen et al., 2011) (Table 2). Both of the D-(+)-glucose:sucrose (molar ratio, 1:1) and maleic acid:choline chloride (molar ratio, 1:3) based DESs could increase the solubility and potentiate the phototoxic effect of the photosensitize (Wikene et al., 2015). In addition, camphor:menthol based DES is considered to be an effective delivery system for the release of ibuprofen more than 7 days (Phaechamud et al., 2016) (Table 2).

## DESS in API Deilivery System for Treatment of Metabolic Diseases

### Cancer

Considering the great mass and energy exchange for cell survival, cancer cells exhibit discernible differences in metabolic level compared to normal cells. They autonomously reprogram their metabolism level and adapt to the tumor microenvironment to meet the vigorous bioenergetic and biosynthetic demand (Martínez-Reyes & Chandel, 2021). At the glucose metabolism level, cancer cells were characterized by high glucose uptake rate, enhanced glycolysis and increased lactic acid production. Even in the nutrient-deprived conditions, cancer cells consume more glucose through glycolysis, but produce less adenosine triphosphate, which is known as the Warburg effect (Xu et al., 2021). Moreover, AKT, mTOR and hypoxia-inducible factor pathways can individually increase glycolysis through transcriptional upregulation and phosphorylation of glucose transporters and glycolytic enzymes, which promote the proliferation of cancer cells (Weng et al., 2020). Therefore, metabolic pathways are attractive therapeutic targets for cancer therapy. For instance, it has been found that limonene induces apoptosis via mitochondrial pathway and affects cell survival/apoptosis via PI3K/Akt signaling pathway in colorectal cancer (Bishnupuri et al., 2019; Araújo-Filho et al., 2021).

Many anti-cancer drugs that target metabolic pathways have been synthesized by the researchers but it remains unsettled to find an economical-friendly and highly soluble solvent for the anti-cancer drugs administration. DESs were applied to the treatment of cancer through their anti-cancer activity or the capacity for dissolving anti-cancer drugs. A limonene based DES (ibuprofen:limonene, with a molar ratio of 1:4) could also effectively inhibit the proliferation of human colon cancer cell line HT29 without affecting the viability of healthy cells, indicating that therapeutic DESs have extensive anti-cancer potential (Pereira et al., 2019) (Table 3). The DES system not only retains the therapeutic effects of limonene and ibuprofen, but also increases the solubility of the two components and reduces the side effect of limonene on the viability of normal cell lines. The specific mechanism may be related to the synergistic or additive effects caused by the hydrogen-bonded supramolecular arrangements between the two components in DESs. α-Chitin nanofibers, prepared from the mixture of choline chloride:urea, choline bromide:urea, or choline chloride:thiourea (Mukesh et al., 2014), can be used to encapsulate calcium alginate into biological nanocomposite gel beads (Table 3). The biomaterial could release the anti-cancer drug 5-fluorouracil under pH 7.4, which proved the ability of DESs as API delivery systems for the treatment of colon cancer, stomach cancer, breast cancer, and other cancers. In addition, Tan and his colleagues synthesized a natural DES on the basis of a binary phase diagram of betaine and mandelic acid to deliver an oral anti-cancer drug (RA-XII). The solubility and oral bioavailability of RA-XII (Liu et al., 2021) were raised up to 17.5 and 11.6 times, respectively, affording an approach for improving the solubility and bioavailability of poorly water-soluble APIs (Table 3).

**TABLE 3:** The composition and proportion of DESs in the treatment of metabolic related diseases.

DESs composition	Molar ratio	API	Diseases	References
Ibuprofen: Limonene	1:4	Limonene	Colon cancer	Pereira et al. (2019)
Choline chloride: Thiourea	1:2	5-Fluorouracil	Multiple cancers	Mukesh et al. (2014)
Choline chloride	2:1	Bis-quinazolin-4-ones	Breast cancer	Ahmed et al. (2019)
L- (+)-tartaric acid			Lung cancer	
Betaine: Mandelic acid	1:1	RA-XII	Multiple cancers	Liu et al. (2021)
Choline chloride: Malic acid	2:1	Insulin	Diabetes mellitus	Li et al. (2019)
Choline chloride: Malic acid	2:1	Insulin	Diabetes mellitus	Vaidya et al. (2020)
Choline chloride: Geranic acid	1:2	Insulin	Diabetes mellitus	Tanner et al. (2018)
Choline bicarbonate: Geranic acid	1:1	Insulin	Diabetes mellitus	Banerjee et al. (2017)
Choline chloride: Glycerol	1:1	Curcumin	Atherosclerosis	Jeliński et al. (2019)
Glucose:Sucrose	1:1	Curcumin	Atherosclerosis	Wikene et al. (2015)

The cytotoxicity of *N,N*-diethylammonium chloride (DAC) based and choline chloride based DES were evaluated by investigating the interaction of DESs and cancer cell lines (HelaS3, AGS, MCF-7 and WRL-68) with the conductor-like screening model for real solvents (Liu et al., 2020; Mbous et al., 2020; Rezaei Motlagh et al., 2020). The results revealed that DAC based DESs (IC50 interval, 37–109 mM) were more toxic than choline chloride based DESs (IC50 interval, 279–1,260 mM), indicating the potential of DAC based DESs as an anti-cancer agent. In addition, the interaction between DESs and cell membrane phospholipids were also investigated using a conductor-like screening model for real solvents (Hayyan et al., 2016). It has been proven that the toxicity mechanism of DESs to the organism or cell is mainly manifested in continuous damage to the plasma membrane, promoting the increase of intracellular reactive oxygen species concentration, causing the oxidative stress cascade reaction, and finally leading to cell apoptosis. Therefore, the safety of DESs can be accessed by cell toxicity assay before application. Besides, choline chloride:tartaric acid based DESs can be used as the reaction medium under ultrasound irradiation for the synthesis of anti-cancer drug (Figure 1, Table 3). Cytotoxic activity of the anti-cancer drug in DESs were investigated with breast (MCF-7) and lung (A549) cancer cell lines (Ahmed Arafa et al., 2019). The results showed that the derivative [3,3'-(sulfonyl (4,1-phenylene)) (2-methyl-6-nitroquinazolin-4 (3)-one)] showed strong inhibitory effect on MCF-7 and A549 cancer cells, but low toxicity to normal breast cell line (MCF-10A).

In general, the cytotoxicity of DESs to normal or cancer cell lines is affected by the composition of hydrogen-bonded donors and receptors. Among them, a hydrogen bond donor is considered the main driving factor of toxicity, while the hydrogen bond receptor has a relatively weak effect on toxicity. In addition, water can significantly reduce the toxicity of ternary natural DESs. Although DES-based polymers and self-assembly drugs have been explored in the treatment of cancer, many efforts still need to be made in transdermal delivery of anti-cancer drugs, synthesis of anti-cancer nanoparticles and safety evaluation *in vivo*.

### Diabetes Mellitus

Diabetes is a group of endocrine diseases characterized by elevated blood sugar, which has become a major safety problem threatening human health. The reduction in glycolysis, pentose phosphate pathway and tricarboxylic acid cycle caused by decreased glucose phosphorylation are the main manifestations of glucose metabolism in diabetes patients (Wu et al., 2018). These manifestations eventually elevate blood sugar and decrease energy supply. In addition, a critical feature in lipid metabolism disorders is the deficiency or insufficiency of insulin, which resulted in the increased plasma concentrations of free fatty acids and triglycerides (Xu et al., 2018).

Insulin has been the most common drug for the treatment of diabetes, especially in type I diabetes and diabetic complications. At present, insulin is mainly administered by subcutaneous injection (Gradel et al., 2020). The invasive operation is not easy to be accepted by the patients. Both clinicians and researchers have searched for a non-invasive method to treat diabetes instead of subcutaneous injection. For instance, DESs have been used as a green alternative solvent to dissolve insulin for the treatment of endocrine diseases through transdermal administration (Vaidya & Mitragotri, 2020; Li et al., 2019). During these treatments, the insulin was mixed with choline:geranic acid based DESs to form a viscoelastic cage gel, which allowed the DES to act as a transport enhancer to continuously release insulin (Vaidya & Mitragotri, 2020) (Figure 1, Table 3). The viscoelastic cage gel exerts the pharmacological effects of insulin by oral administration and reduces blood glucose levels in a dose-dependent manner, confirming the feasibility of ILs/DESs as drug carriers in the treatment of diabetes. Furthermore, the hypoglycemic effect of insulin solution, insulin-hydrogel, and insulin-DESs (choline chloride:malic acid) was observed by nasal administration (Li et al., 2019) (Table 3). Insulin-DESs have been proven to be superior to insulin-hydrogel and insulin solutions, showing the potential of DESs as insulin carriers for diabetes therapy.

Transdermal administration of choline:geranic acid based DESs can also increase API delivery efficiency for insulin and increase the skin permeability of the drug (Banerjee, et al., 2017; Tanner, et al., 2018) (Table 3). Topical administration of insulin dispersed DESs (25 U kg^−1^ insulin dose) significantly reduced blood glucose levels within 4 hours in a time-dependent manner. In brief, DESs can be used as a promising insulin carrier in the treatment of endocrine diabetes, which is administered through the skin, nasal mucosa or oral mucosa.

### Atherosclerosis

Atherosclerosis, one of the most common cardiovascular disorders is highly associated with the disorders of lipid and lipoprotein metabolism (Flora & Nayak, 2019). Curcumin could protect against arterial damage by improving serum lipid levels (Li et al., 2015). In consideration of low solubility and bioavailability of curcumin, choline chloride:glycerol based DESs has been developed as API delivery systems by Jeliński and his coworkers (Jeliński et al., 2019) (Table 3). Curcumin solubility in the DES increase to 7.25 mg/g, compared with 0.0006 mg/g in water. In addition, Wikene and his colleagues prepared D-(+)-glucose:sucrose and maleic acid/choline chloride based DES and assessed the potential of the DESs as the solvent for curcumin in antimicrobial photodynamic therapy (Wikene et al., 2015) (Table 3). They found that the DESs can lock the photo sensitizer within one specific molecular conformation and potentiate its phototoxic effect, demonostrating the unique properties of the DESs as the solvents.

## Conclusions

As green, economical and biodegradable solvents, DESs are becoming increasingly important because of their abundant precursor components, accessible synthesis methodologies and potential to improve drug solubility and bioavailability. DESs have been effectively employed as API delivery systems in the treatment of metabolic related diseases. However, it is difficult to achieve long-term sustained release using most DESs carriers. Additional efforts should be done to develop the sustained DESs delivery systems, assess their safety *in vivo*, and explore their effectiveness in metabolic-related diseases. Moreover, substantial work should be done in the clinical treatment of metabolic diseases using DES-based API delivery systems.

## References

[B1] Ahmed Arafa W. A. (2019). Deep Eutectic Solvent for an Expeditious Sono-Synthesis of Novel Series of Bis-Quinazolin-4-One Derivatives as Potential Anti-cancer Agents. R. Soc. Open. Sci. 6, 182046. 10.1098/rsos.182046 31032048 PMC6458391

[B2] Alizadeh V. Malberg F. Pádua A. A. H. Kirchner B. (2020). Are There Magic Compositions in Deep Eutectic Solvents? Effects of Composition and Water Content in Choline Chloride/Ethylene Glycol from Ab Initio Molecular Dynamics. J. Phys. Chem. B. 124, 7433–7443. 10.1021/acs.jpcb.0c04844 32790407

[B3] Araújo-Filho H. G. Dos Santos J. F. Carvalho M. T. B. Picot L. Fruitier-Arnaudin I. Groult H. (2021). Anticancer Activity of Limonene: A Systematic Review of Target Signaling Pathways. Phytother. Res. 35, 4957–4970. 10.1002/ptr.7125 33864293

[B4] Aroso I. M. Craveiro R. Rocha Â. Dionísio M. Barreiros S. Reis R. L. (2015). Design of Controlled Release Systems for THEDES-Therapeutic Deep Eutectic Solvents, Using Supercritical Fluid Technology. Int. J. Pharm. 492, 73–79. 10.1016/j.ijpharm.2015.06.038 26142248

[B5] Aroso I. M. Silva J. C. Mano F. Ferreira A. S. Dionísio M. Sá-Nogueira I. (2016). Dissolution Enhancement of Active Pharmaceutical Ingredients by Therapeutic Deep Eutectic Systems. Eur. J. Pharm. Biopharm. 98, 57–66. 10.1016/j.ejpb.2015.11.002 26586342

[B6] Banerjee A. Ibsen K. Iwao Y. Zakrewsky M. Mitragotri S. (2017). Transdermal Protein Delivery Using Choline and Geranate (CAGE) Deep Eutectic Solvent. Adv. Healthc. Mater. 6, 15. 10.1002/adhm.201601411 28337858

[B7] Bishnupuri K. S. Alvarado D. M. Khouri A. N. Shabsovich M. Chen B. Dieckgraefe B. K. (2019). Ido1 and Kynurenine Pathway Metabolites Activate PI3K-Akt Signaling in the Neoplastic Colon Epithelium to Promote Cancer Cell Proliferation and Inhibit Apoptosis. Cancer Res. 79, 1138–1150. 10.1158/0008-5472.Can-18-0668 30679179 PMC6420842

[B8] Boroughs L. K. DeBerardinis R. J. (2015). Metabolic Pathways Promoting Cancer Cell Survival and Growth. Nat. Cell Biol. 17, 351–359. 10.1038/ncb3124 25774832 PMC4939711

[B9] Chen M. Zhang X. Wang H. Lin B. Wang S. Hu G. (2015). Determination of Rutin in Rat Plasma by Ultra Performance Liquid Chromatography Tandem Mass Spectrometry and Application to Pharmacokinetic Study. J. Chromatogr. Sci. 53, 519–525. 10.1093/chromsci/bmu078 25030991

[B10] Cordeiro T. Castiñeira C. Mendes D. Danède F. Sotomayor J. Fonseca I. M. (2017). Stabilizing Unstable Amorphous Menthol through Inclusion in Mesoporous Silica Hosts. Mol. Pharm. 14, 3164–3177. 10.1021/acs.molpharmaceut.7b00386 28836790

[B11] De Jong W. H. Borm P. J. (2008). Drug Delivery and Nanoparticles:Applications and Hazards. Int. J. Nanomedicine 3, 133–149. 10.2147/ijn.s596 18686775 PMC2527668

[B12] Duarte A. R. Ferreira A. S. Barreiros S. Cabrita E. Reis R. L. Paiva A. (2017). A Comparison between Pure Active Pharmaceutical Ingredients and Therapeutic Deep Eutectic Solvents: Solubility and Permeability Studies. Eur. J. Pharm. Biopharm. 114, 296–304. 10.1016/j.ejpb.2017.02.003 28189620

[B13] Edupuganti O. P. Ovsepian S. V. Wang J. Zurawski T. H. Schmidt J. J. Smith L. (2012). Targeted Delivery into Motor Nerve Terminals of Inhibitors for SNARE-Cleaving Proteases via Liposomes Coupled to an Atoxic Botulinum Neurotoxin. Febs. J. 279, 2555–2567. 10.1111/j.1742-4658.2012.08638.x 22607388

[B14] Faggian M. Sut S. Perissutti B. Baldan V. Grabnar I. Dall'Acqua S. (2016). Natural Deep Eutectic Solvents (NADES) as a Tool for Bioavailability Improvement: Pharmacokinetics of Rutin Dissolved in Proline/Glycine after Oral Administration in Rats: Possible Application in Nutraceuticals. Molecules 21, 1531. 10.3390/molecules21111531 PMC6272970 27854256

[B15] Flora G. D. Nayak M. K. (2019). A Brief Review of Cardiovascular Diseases, Associated Risk Factors and Current Treatment Regimes. Curr. Pharm. Des. 25, 4063–4084. 10.2174/1381612825666190925163827 31553287 PMC12994374

[B16] Gala U. Chuong M. C. Varanasi R. Chauhan H. (2015). Characterization and Comparison of Lidocaine-Tetracaine and Lidocaine-Camphor Eutectic Mixtures Based on Their Crystallization and Hydrogen-Bonding Abilities. AAPS. Pharmscitech. 16, 528–536. 10.1208/s12249-014-0242-4 25370024 PMC4444629

[B17] Gradel A. K. J. Porsgaard T. Lykkesfeldt J. Brockhoff P. B. Seested T. Refsgaard H. H. F. (2020). Subcutaneous Administration of Insulin Is Associated with Regional Differences in Injection Depot Variability and Kinetics in the Rat. Exp. Clin. Endocrinol. Diabetes 128, 332–338. 10.1055/a-0658-1089 30075480

[B18] Gurkan B. Squire H. Pentzer E. (2019). Metal-Free Deep Eutectic Solvents: Preparation, Physical Properties, and Significance. J. Phys. Chem. Lett. 10, 7956–7964. 10.1021/acs.jpclett.9b01980 31804088

[B19] Hayyan M. Mbous Y. P. Looi C. Y. Wong W. F. Hayyan A. Salleh Z. (2016). Natural Deep Eutectic Solvents: Cytotoxic Profile. Springerplus 5, 913. 10.1186/s40064-016-2575-9 27386357 PMC4927554

[B20] Hoffman D. J. Powell T. L. Barrett E. S. Hardy D. B. (2021). Developmental Origins of Metabolic Diseases. Physiol. Rev. 101 (3), 739–795. 10.1152/physrev.00002.2020 33270534 PMC8526339

[B21] Jeliński T. Przybyłek M. Cysewski P. (2019). Natural Deep Eutectic Solvents as Agents for Improving Solubility, Stability and Delivery of Curcumin. Pharm. Res. 36, 116. 10.1007/s11095-019-2643-2 31161340 PMC6546644

[B22] Kane A. E. Sinclair D. A. (2018). Sirtuins and NAD+ in the Development and Treatment of Metabolic and Cardiovascular Diseases. Circ. Res. 123, 868–885. 10.1161/circresaha.118.312498 30355082 PMC6206880

[B23] Kaplun-Frischoff Y. Touitou E. (1997). Testosterone Skin Permeation Enhancement by Menthol through Formation of Eutectic with Drug and Interaction with Skin Lipids. J. Pharm. Sci. 86, 1394–1399. 10.1021/js9701465 9423153

[B24] Li W. Wang L. Huang W. Skibba M. Fang Q. Xie L. (2015). Inhibition of ROS and Inflammation by an Imidazopyridine Derivative X22 Attenuate High Fat Diet-Induced Arterial Injuries. Vascul. Pharmacol. 72, 153–162. 10.1016/j.vph.2015.05.006 25989105

[B25] Li Y. Wu X. Zhu Q. Chen Z. Lu Y. Qi J. (2019). Improving the Hypoglycemic Effect of Insulin via the Nasal Administration of Deep Eutectic Solvents. Int. J. Pharm. 569, 118584. 10.1016/j.ijpharm.2019.118584 31376466

[B26] Li Z. Lee P. I. (2016). Investigation on Drug Solubility Enhancement Using Deep Eutectic Solvents and Their Derivatives. Int. J. Pharm. 505 (1-2), 283–288. 10.1016/j.ijpharm.2016.04.018 27079143

[B27] Liu M. Lai Z. Zhu L. Ding X. Tong X. Wang Z. (2021). Novel Amorphous Solid Dispersion Based on Natural Deep Eutectic Solvent for Enhancing Delivery of Anti-tumor RA-XII by Oral Administration in Rats. Eur. J. Pharm. Sci. 166, 105931. 10.1016/j.ejps.2021.105931 34256100

[B28] Liu Y. Friesen J. B. McAlpine J. B. Lankin D. C. Chen S. N. Pauli G. F. (2018). Natural Deep Eutectic Solvents: Properties, Applications, and Perspectives. J. Nat. Prod. 81, 679–690. 10.1021/acs.jnatprod.7b00945 29513526 PMC5913660

[B29] Liu Y. Yu H. Sun Y. Zeng S. Zhang X. Nie Y. (2020). Screening Deep Eutectic Solvents for CO2 Capture with COSMO-RS. Front. Chem. 8, 82. 10.3389/fchem.2020.00082 32117899 PMC7031488

[B30] Liu Y. Zhang H. Yu H. Guo S. Chen D. (2019). Deep Eutectic Solvent as a Green Solvent for Enhanced Extraction of Narirutin, Naringin, Hesperidin and Neohesperidin from Aurantii Fructus. Phytochem. Anal. 30, 156–163. 10.1002/pca.2801 30426588

[B31] Lodzki M. Godin B. Rakou L. Mechoulam R. Gallily R. Touitou E. (2003). Cannabidiol-Transdermal Delivery and Anti-inflammatory Effect in a Murine Model. J. Control Release. 93, 377–387. 10.1016/j.jconrel.2003.09.001 14644587

[B32] Makoś P. Fernandes A. Przyjazny A. Boczkaj G. (2018). Sample Preparation Procedure Using Extraction and Derivatization of Carboxylic Acids from Aqueous Samples by Means of Deep Eutectic Solvents for Gas Chromatographic-Mass Spectrometric Analysis. J. Chromatogr. A. 1555, 10–19. 10.1016/j.chroma.2018.04.054 29705647

[B33] Mano F. Martins M. Sá-Nogueira I. Barreiros S. Borges J. P. Reis R. L. (2017). Production of Electrospun Fast-Dissolving Drug Delivery Systems with Therapeutic Eutectic Systems Encapsulated in Gelatin. AAPS. Pharmscitech. 18, 2579–2585. 10.1208/s12249-016-0703-z 28236268

[B34] Martínez-Reyes I. Chandel N. S. (2021). Cancer Metabolism: Looking Forward. Nat. Rev. Cancer 21, 669–680. 10.1038/s41568-021-00378-6 34272515

[B35] Mbous Y. P. Hayyan M. Wong W. F. Hayyan A. Looi C. Y. Hashim M. A. (2020). Simulation of Deep Eutectic Solvents' Interaction with Membranes of Cancer Cells Using COSMO-RS. J. Phys. Chem. B. 124, 9086–9094. 10.1021/acs.jpcb.0c04801 32930594

[B36] Morimoto Y. Hayashi T. Kawabata S. Seki T. Sugibayashi K. (2000). Effect of L-Menthol-Ethanol-Water System on the Systemic Absorption of Flurbiprofen after Repeated Topical Applications in Rabbits. Biol. Pharm. Bull. 23, 1254–1257. 10.1248/bpb.23.1254 11041263

[B37] Morrison H. G. Sun C. C. Neervannan S. (2009). Characterization of Thermal Behavior of Deep Eutectic Solvents and Their Potential as Drug Solubilization Vehicles. Int. J. Pharm. 378, 136–139. 10.1016/j.ijpharm.2009.05.039 19477257

[B38] Mukesh C. Mondal D. Sharma M. Prasad K. (2014). Choline Chloride-Thiourea, a Deep Eutectic Solvent for the Production of Chitin Nanofibers. Carbohydr. Polym. 103, 466–471. 10.1016/j.carbpol.2013.12.082 24528755

[B39] Park C. W. Kim J. Y. Rhee Y. S. Oh T. O. Ha J. M. Choi N. Y. (2012). Preparation and Valuation of a Topical Solution Containing Eutectic Mixture of Itraconazole and Phenol. Arch. Pharm. Res. 35, 1935–1943. 10.1007/s12272-012-1110-y 23212635

[B40] Pearson E. R. (2019). Type 2 Diabetes: a Multifaceted Disease. Diabetologia 62, 1107–1112. 10.1007/s00125-019-4909-y 31161345 PMC6560016

[B41] Pena-Pereira F. Namieśnik J. (2014). Ionic Liquids and Deep Eutectic Mixtures: Sustainable Solvents for Extraction Processes. ChemSusChem 7, 1784–1800. 10.1002/cssc.201301192 24811900

[B42] Pereira C. V. Silva J. M. Rodrigues L. Reis R. L. Paiva A. Duarte A. R. C. (2019). Unveil the Anticancer Potential of Limomene Based Therapeutic Deep Eutectic Solvents. Sci. Rep. 9, 14926. 10.1038/s41598-019-51472-7 31624310 PMC6797721

[B43] Phaechamud T. Tuntarawongsa S. Charoensuksai P. (2016). Evaporation Behavior and Characterization of Eutectic Solvent and Ibuprofen Eutectic Solution. AAPS. Pharmscitech. 17, 1213–1220. 10.1208/s12249-015-0459-x 26669887

[B69] Poznyak A. Grechko A. V. Poggio P. Myasoedova V. A. Alfieri V. Orekhov A. N. (2020). The Diabetes Mellitus-Atherosclerosis Connection: The Role of Lipid and Glucose Metabolism and Chronic Inflammation. Int. J. Mol. Sci. 21, 1835. 10.3390/ijms21051835 PMC7084712 32155866

[B44] Rezaei Motlagh S. Harun R. Awang Biak D. R. Hussain S. A. Omar R. Elgharbawy A. A. (2020). COSMO-RS Based Prediction for Alpha-Linolenic Acid (ALA) Extraction from Microalgae Biomass Using Room Temperature Ionic Liquids (RTILs). Mar. Drugs 18, 108. 10.3390/md18020108 PMC7074282 32059424

[B45] Saha M. Rahman M. S. Hossain M. N. Raynie D. E. Halim M. A. (2020). Molecular and Spectroscopic Insights of a Choline Chloride Based Therapeutic Deep Eutectic Solvent. J. Phys. Chem. A. 124, 4690–4699. 10.1021/acs.jpca.0c00851 32396354

[B46] Sangtarashani S. M. H. Rahmaninia M. Behrooz R. Khosravani A. (2020). Lignocellulosic Hydrogel from Recycled Old Corrugated Container Resources Using Ionic Liquid as a Green Solvent. J. Environ. Manage. 270, 110853. 10.1016/j.jenvman.2020.110853 32501240

[B47] Sekiguchi K. Obi N. Ueda Y. (1964). Studies on Absorption of Eutectic Mixture. II. Absorption of Fused Conglomerates of Chloramphenicol and Urea in Rabbits. Chem. Pharm. Bull. (Tokyo) 12, 134–144. 10.1248/cpb.12.134 14126741

[B48] Shekaari H. Zafarani-Moattar M. T. Mokhtarpour M. (2017). Solubility, Volumetric and Compressibility Properties of Acetaminophen in Some Aqueous Solutions of Choline Based Deep Eutectic Solvents at T=(288.15 to 318.15) K. Eur. J. Pharm. Sci. 109, 121–130. 10.1016/j.ejps.2017.07.021 28739326

[B49] Shen Q. Li X. Li W. Zhao X. (2011). Enhanced Intestinal Absorption of Daidzein by Borneol/Menthol Eutectic Mixture and Microemulsion. AAPS. Pharmscitech. 12, 1044–1049. 10.1208/s12249-011-9672-4 21842308 PMC3225554

[B50] Shi S. Kong N. Feng C. Shajii A. Bejgrowicz C. Tao W. (2019). Drug Delivery Strategies for the Treatment of Metabolic Diseases. Adv. Healthc. Mater. 8, e1801655. 10.1002/adhm.201801655 30957991 PMC6663576

[B51] Silva J. M. Pereira C. V. Mano F. Silva E. Castro V. I. B. Sá-Nogueira I. (2019). Therapeutic Role of Deep Eutectic Solvents Based on Menthol and Saturated Fatty Acids on Wound Healing. ACS. Appl. Bio. Mater. 2, 4346–4355. 10.1021/acsabm.9b00598 PMC6993812 32030369

[B52] Stott P. W. Williams A. C. Barry B. W. (1998). Transdermal Delivery from Eutectic Systems: Enhanced Permeation of a Model Drug, Ibuprofen. J. Control Release. 50, 297–308. 10.1016/s0168-3659(97)00153-3 9685897

[B53] Sut S. Faggian M. Baldan V. Poloniato G. Castagliuolo I. Grabnar I. (2017). Natural Deep Eutectic Solvents (NADES) to Enhance Berberine Absorption: An *In Vivo* Pharmacokinetic Study. Molecules 22, 1921. 10.3390/molecules22111921 PMC6150298 29117131

[B54] Tanner E. E. L. Ibsen K. N. Mitragotri S. (2018). Transdermal Insulin Delivery Using Choline-Based Ionic Liquids (CAGE). J. Control Release 286, 137–144. 10.1016/j.jconrel.2018.07.029 30026081

[B55] Taylor R. Al-Mrabeh A. Zhyzhneuskaya S. Peters C. Barnes A. C. Aribisala B. S. (2018). Remission of Human Type 2 Diabetes Requires Decrease in Liver and Pancreas Fat Content but Is Dependent upon Capacity for β Cell Recovery. Cell. Metab. 28, 547–e3. 10.1016/j.cmet.2018.07.003 30078554

[B56] Tian A. P. Yin Y. K. Yu L. Yang B. Y. Li N. Li J. Y. (2020). Topical Delivery of Modified Da-Cheng-Qi Decoction () Using Low-Frequency Ultrasound Sonophoresis for Refractory Metastatic Malignant Bowel Obstruction: An Open-Label Single-Arm Clinical Trial. Chin. J. Integr. Med. 26, 382–387. 10.1007/s11655-019-3041-7 31134466

[B57] Vaidya A. Mitragotri S. (2020). Ionic Liquid-Mediated Delivery of Insulin to Buccal Mucosa. J. Control Release 327, 26–34. 10.1016/j.jconrel.2020.07.037 32735879

[B58] Wang W. Cai Y. Liu Y. Zhao Y. Feng J. Liu C. (2017). Microemulsions Based on Paeonol-Menthol Eutectic Mixture for Enhanced Transdermal Delivery: Formulation Development and *In Vitro* Evaluation. Artif. Cell Nanomed. Biotechnol. 45, 1–6. 10.1080/21691401.2016.1226178 27600884

[B59] Weng M. L. Chen W. K. Chen X. Y. Lu H. Sun Z. R. Yu Q. (2020). Fasting Inhibits Aerobic Glycolysis and Proliferation in Colorectal Cancer via the Fdft1-Mediated AKT/mTOR/HIF1α Pathway Suppression. Nat. Commun. 11, 1869. 10.1038/s41467-020-15795-8 32313017 PMC7170903

[B60] Wikene K. O. Bruzell E. Tønnesen H. H. (2015). Characterization and Antimicrobial Phototoxicity of Curcumin Dissolved in Natural Deep Eutectic Solvents. Eur. J. Pharm. Sci. 80, 26–32. 10.1016/j.ejps.2015.09.013 26410725

[B61] Wojnarowska Z. Smolka W. Zotova J. Knapik-Kowalczuk J. Sherif A. Tajber L. (2018). The Effect of Electrostatic Interactions on the Formation of Pharmaceutical Eutectics. Phys. Chem. Chem. Phys. 20, 27361–27367. 10.1039/c8cp05905e 30357184

[B62] Woolfson A. D. Malcolm R. K. Campbell K. Jones D. S. Russell J. A. (2000). Rheological, Mechanical and Membrane Penetration Properties of Novel Dual Drug Systems for Percutaneous Delivery. J. Control Release. 67, 395–408. 10.1016/s0168-3659(00)00230-3 10825570

[B63] Wu D. Hu D. Chen H. Shi G. Fetahu I. S. Wu F. (2018). Glucose-Regulated Phosphorylation of TET2 by AMPK Reveals a Pathway Linking Diabetes to Cancer. Nature 559, 637–641. 10.1038/s41586-018-0350-5 30022161 PMC6430198

[B64] Xin Y. Huang M. Guo W. W. Huang Q. Zhang L. Z. Jiang G. (2017). Nano-based Delivery of RNAi in Cancer Therapy. Mol. Cancer 16, 134. 10.1186/s12943-017-0683-y 28754120 PMC5534073

[B65] Xu K. Yin N. Peng M. Stamatiades E. G. Shyu A. Li P. (2021). Glycolysis Fuels Phosphoinositide 3-Kinase Signaling to Bolster T Cell Immunity. Science 371, 405–410. 10.1126/science.abb2683 33479154 PMC8380312

[B66] Xu L. Li Y. Yin L. Qi Y. Sun H. Sun P. (2018). MiR-125a-5p Ameliorates Hepatic Glycolipid Metabolism Disorder in Type 2 Diabetes Mellitus through Targeting of STAT3. Theranostics 8, 5593–5609. 10.7150/thno.27425 30555566 PMC6276304

[B67] Zainal-Abidin M. H. Hayyan M. Ngoh G. C. Wong W. F. (2020). Doxorubicin Loading on Functional Graphene as a Promising Nanocarrier Using Ternary Deep Eutectic Solvent Systems. ACS. Omega. 5, 1656–1668. 10.1021/acsomega.9b03709 32010840 PMC6990633

[B68] Zakrewsky M. Banerjee A. Apte S. Kern T. L. Jones M. R. Sesto R. E. (2016). Choline and Geranate Deep Eutectic Solvent as a Broad-Spectrum Antiseptic Agent for Preventive and Therapeutic Applications. Adv. Healthc. Mater. 5, 1282–1289. 10.1002/adhm.201600086 26959835

